# Rare Diseases: Implementation of Molecular Diagnosis, Pathogenesis Insights and Precision Medicine Treatment

**DOI:** 10.3390/ijms24109064

**Published:** 2023-05-22

**Authors:** Lidia Larizza, Maria Vittoria Cubellis

**Affiliations:** 1 Experimental Research Laboratory of Medical Cytogenetics and Molecular Genetics, IRCCS Istituto Auxologico Italiano, Via Ariosto 13, 20145 Milan, Italy; 2 Dipartimento di Biologia, Università Federico II, 80126 Naples, Italy; cubellis@unina.it; 3 Biology and Evolution of Marine Organisms, Stazione Zoologica Anton Dohrn, Villa Comunale, 80121 Naples, Italy; 4 Istituto di Chimica Biomolecolare—CNR, 80078 Pozzuoli, Italy

Rare Diseases (RD) do not have an exact definition since local authorities define the criteria in different ways, from fewer than 5 people in 10,000, according to the European Union, to the standard world average of 40 cases per 100,000 people [1]. The number of recognized orphan diseases is usually around 7000, ranging from 5000 to 8000 according to different definitions [2]. Despite their huge number and variability, RD share difficulties related to (i) diagnostic delay and the prolonged patient odyssey; (ii) lack/scarcity of disease biomarkers providing insights into the pathogenesis; and (iii) challenging drug discovery and treatment [3,4]. However, the advances in the last decade in Whole Exome Sequencing (WES) and Whole Genome Sequencing (WGS), using a next generation sequencing (NGS) platform, have revolutionized the approach to RD, yielding previously unimaginable diagnoses and gene discoveries in 25–50% of unsolved patients [5]. The first report using WES for diagnosing Miller syndrome in 2010 [6] represents a game-changer [7] in the transformation of the approach to RD. Promising avenues for therapy were disclosed by preclinical studies on mouse- and patients-induced pluripotent stem cell (iPSC) in vitro models and genome editing. Precision medicine therapeutics became increasingly tractable to a small numbers of RD patients’ as their captured genetics was thought might potentially open the path toward identifying therapies for common diseases patients [8]. 

The rapidly changing landscape of RD requires us, periodically, to update diagnostic omics and bioinformatics workflows of known and newly discovered clinical entities as well as the roadmaps for tailored patient treatment. 

These challenging issues paved the way to RDMMTS Special Issue IV, which includes 12 contributions—9 research and 3 review articles—focusing on different RD. 

In a few rare diseases, such as juvenile angiofibromas (JA), diagnostics and treatment are at an advanced stage, but etiopathogenesis is controversial. The study by Schick et al. [9] fills this gap, highlighting that these benign tumors, most frequent in adolescent males, are a malformation arising from the neural crest cells of the first branchial arch plexus. Evidence is provided by positive immunohistochemistry of the neural stem cell marker CD271 in a series of 22 JA and by the quantitative expression profile of Epithelial Mesenchymal Transition (EMT) markers, accounting for JA fibrous and vascular composition, in CD271-positive and CD271-negative cells derived from one tumor.

The diagnostic and therapeutic aspects are at the root of the paper by Magri and co-workers, which describes a patient affected by limb-girdle muscular dystrophy (LGMD) caused by molecular defects in the SGCB gene [10]. They report the early molecular diagnosis of this almost-asymptomatic patient and the consequent diagnosis of a patient with similar clinical features deceased more than twenty years earlier. Identifying a defect that alters a splicing site allowed the authors to test the feasibility of morpholino-based antisense oligomers, which resulted in the complete correction of aberrant splicing and an increase in normal transcripts. In vitro experiments were promising and paved the way towards expanding morpholino-based antisense oligomers, currently approved for Duchenne muscular dystrophy and Huntington’s disease.

The generation of iPSC-neurons from patients with neurodevelopmental diseases has provided an in vitro platform to test known drugs and small molecules able to rescue or attenuate disease biomarkers [11] or to screen myriad small molecules. Taking into account the clinical heterogeneity of Rett (RTT) syndrome, Perego et al. [12] modeled and characterized iPSC-neurons from patients representative of the RTT spectrum, including girls with MECP2 “hot spot” variants associated with mild and severe phenotypes and a hemizygous male. Truncated MeCP2 proteins were revealed by Western blot and immunofluorescence analysis in the cells from the male and the seriously affected girls, consistent with the patients’ clinical presentation. Characterization of RTT iPSC-neurons, including isogenic controls from females, as compared to neurons from control individuals, evidenced reduced nuclear size and branch number in differentiating neurons and variably immature firing and voltage currents in patch-clamp mature neurons. Interestingly, the morphological and electric activity biomarkers mirror the clinical severity of the donor patients, indicating that the in vitro neuronal model is suitable to investigate drug effects on the cellular phenotype for translational purposes, as shown on iPSC neurons from other epigenetic neurodevelopmental disorders [11]

The article by Grosse et al. [13] adds the clinical and molecular characterizations of three novel patients to the recently described intellectual disability syndrome associated with mild dysmorphisms caused by defective activity of SOX4. [14]. SOX4 (OMIM*184430) is a transcription factor which, by interacting with other transcription factors, exerts pleiotropic functions in corticogenesis. Functional studies of patients’ variants, performed by co-expressing mutant SOX4 with its coactivator and measuring their activity in reporter assays, demonstrated abolished SOX4 activity in all cases. As well as providing insights into the pathogenesis of the SOX4-related neurodevelopmental syndrome, the authors point out the incomplete penetrance associated with one characterized variant which was inherited by a mildly affected parent; this finding will improve the classification of novel, likely pathogenic, SOX4 variants. 

The study by Colombo et al. [15] attests to how combined WES and molecular modeling can reduce the diagnostic gap of RD, leading to the discovery of novel ultra-rare diseases. Whole exome sequencing of two siblings presenting a phenotype reminiscent of Rothmund Thomson syndrome (RTS) [16] disclosed two linked homozygous missense variants, inherited from healthy first cousin parents, in exons 3 and 20 of the nucleoporin 98 (NUP98) gene. NUP98 belongs to the group of “dynamic” Nups characterized by multiple FG (Phenylalananine-Glycine) repeats and a high content of intrinsically disordered regions (IDRs) [17]. These FG-Nups shuttle off the nuclear pore to the nucleoplasm, where they interact with transcription factors and chromatin modifiers. The higher pathogenic score and key position between FG repeats of the amino acid replaced by exon 3 variant prompted the authors to characterize the FG domain of the mutant protein by molecular dynamics simulation and to follow up its conformational motion over time with respect to the wild-type FG domain. This study evidenced in the mutated FG domain a reduction in cohesiveness, which influenced its folding and compromised its role as a multi-docking station for proteins and RNA and a hub for the recruitment of chromatin and nuclear factors needed for the maintenance of genomic stability. The clinical overlap of *NUP98*-mutated and RTS patients, indicated by converging dysregulated gene networks, supports this first-described NUP98 nucleoporopathy, adding to the list of inherited NUPs disorders and expanding the well-known role of NUP98 in cancer.

The issue of the scarcity of reliable biomarkers acting as a bridge towards RD diagnosis and the search of therapies is adequately addressed by Delgadillo and coworkers [18], who report two patients clinically diagnosed as affected by the autoimmune lymphoproliferative syndrome (ALPS) characterized by alterations in the lymphocyte apoptotic pathway and associated with autoimmunity. The authors screened for mutations usually involving *Fas*, *FasL* and *Casp10* genes and detected, only in patient 2, a *Fas* variant, resulting in an in-frame premature truncation at a highly conserved protein domain, previously reported in an APLS patient [19]. To look deeper into the cause of disease in patient 1, they further investigated the undiagnosed patient via proteomics. This analysis allowed them to identify differences in the level of expression of proteins involved in the regulation of the cell cycle and immune response. However, the most intriguing result was the identification of EF27, a transcription factor regulating cell cycle, apoptosis, differentiation and senescence, deregulated in some tumours, as the most under expressed within the small group of downregulated proteins. This finding is likely at the root of the disease in patient 1, increasing the knowledge of APLS and opening new scenarios for diagnosis and the development of new therapeutic approaches.

Drug discovery is a significant issue for rare diseases, especially considering financial investments required for de novo discovery. Funding is limited due to the lack of attention by pharmaceutical industries and public investments [20,21]. In this context, the so-called drug repositioning is a helpful tool since it reduces the risk of failure from more than 95% to around 45% compared to *de novo* discovery [22,23]. The contribution of RD-MMTS IV in this field was addressed mainly to lysosomal storage disorders (LSDs), representing a large group of diseases caused by mutations in the genes encoding lysosomal proteins (mainly enzymes).

The paper by Monticelli and co-workers relies on a described cell model, which had previously allowed the authors to identify the role of acetylsalicylic acid in potentiating the approved pharmacological chaperone therapy for Fabry Disease (FD) [24]. In this work, they focused on repurposing a notorious nutraceutical, curcumin, and described its capability to increase the alpha-galactosidase (AGAL) activity by increasing intracellular protein [25]. Interestingly, they demonstrated the stand-alone role of curcumin and the possibility of enhancing the effects of pharmacological chaperones in combined therapies. The effects of drugs were also analyzed at a phenotypic level, visualizing the reduction in the substrate storage and the reduced expression of lysosomal enzymes other than AGAL in long-term treatments.

The perspective of combining drugs to optimize therapies underlies the paper by Iacobucci and co-workers, which focuses on enzyme replacement therapy (ERT) for FD [26]. Their paper is structured to provide a proof-of-concept of two approaches to optimizing ERT. Firstly, they describe the benefits of combined administration of the recombinant enzyme with galactose, which acts as a pharmacological chaperone and prolongs the enzyme’s half-life. Then, they explore the identification of additional targets through proteomics experiments, leading to the discovery of intracellular AGAL protein–protein interactions for wild-type and recombinant enzymes. Such a list of interactors serves as a foundation for repositioning approved drugs, helping to increase the efficacy of recombinant enzyme administration. The authors also comment on the necessity of further deepening the intracellular interactions and the possible effects of commonly used drugs targeting these interactors, which could reveal themselves as reducers rather than adjuvants. In fact, besides the stability extension, they could, unpleasantly, cause a faster degradation or secretion.

Drugs can act by different, unpredictable mechanisms, and the cellular context usually has a primary role in the response. This consideration represented the core of the work described in the paper by Pantoom and co-workers, who focused their attention on the mechanism of ambroxol in increasing β-glucosidase, the enzyme mutated in Gaucher Disease (GD) [27]. Ambroxol is a drug which has been repurposed from its canonical use to treat cystic fibrosis to GD, and its effects were initially related to a pharmacological chaperoning activity. In this paper, the authors describe an insight into the molecular mechanism of action, looking into the in vitro effects on the pure protein and GD cell models. Their data provide new evidence that ambroxol does not act directly on the protein; rather, the mode of its activity, in a cellular context, remains unexplained.

Three review articles complete RDPMTSIV. The review by De Ponti et al. [28] comprehensively delineates the pathomechanisms of Mucopolysaccharidosis type I (MPSI) (OMIM #252800), caused by biallelic pathogenic variants of the *IDUA* gene and deficit of the lysosomal alpha-L-iduronidase enzyme, accumulation of glycosaminoglycans (GAGs) in the lysosomes, lysosomes dysfunction and multisystemic disease. The timeline clinical manifestations of severe MPS1 are described with special focus on the constellation of skeletal abnormalities (“Dysostosis Multiplex”) which do not benefit even from the therapeutic option of donor hematopoietic stem cell transplantation (HSCT) due to poor enzyme diffusion in this anatomical compartment. Enzyme replacement therapy (ERT) of the human recombinant IDUA, approved by the FDA in 2003 and considered the standard of care, proved of benefit to precociously treated patients, but only when combined to HSCT did it improve the outcomes of skeletal manifestations. Gene therapy and gene editing approaches are promising options, mainly investigated on mouse models, but few clinical applications have been tested in MPSI patients. Alternative approaches—including (i) blocking GAG production instead than promoting their catabolism; (ii) suppressing non sense mediate mRNA decay in case of nonsense mutations to bypass the termination codon; and (iii) adopting the chaperone option in case of mutations that impair protein folding, trafficking and accumulation—are explored in mouse models, but the applicability to patients is limited. Given that the skeletal issues, often refractory to the current therapeutic strategies, are crucial in determining the disease severity and the quality of life of MPSI patients, the authors conclude that the pathomechanism underlying impaired bone development and ossification should be better dissected to design tailored therapies to be administered as early as possible to MPSI children. 

The review article on Schuurs–Hoeijmakers Syndrome or PACS1 (phosphofurin acidic cluster sorting protein 1) neurodevelopmental disorder (PACS1-NDD) (OMIM 615009 [29] underlines this syndrome as a paradigm for therapeutic approaches. Indeed, despite the clinical severity (intellectual disability, epilepsy, autism, feeding problems, distinctive craniofacial anomalies) ranges across patients, intriguingly, most of them harbor a recurrent de novo *PACS1* missense mutation (c.607C > T) (p.R203W), hypothesized to act by gain-of-function (GOF) or a dominant negative mechanism. Different therapies that prevent accumulation of the mutant protein have been developed—first of all antisense oligonucleotides (ASO), targeting the mutant mRNAs for degradation, though ASO are incapable of crossing the blood–brain barrier and require direct central nervous system administration. The role of PACS1 in regulating membrane trafficking through the interaction with a number of client proteins, whose function is altered in the patients, fueled a patent application in which fibroblasts from patients with a stronger binding of mutated PACS1 to Histone Deacetylase 6 (HDAC6) revert to the normal cellular phenotype upon treatment with wide-spectrum or specific HDAC6 inhibitors [30]. Other approaches, such as the proteolysis-targeted chimeras’ (PROTACS) selective targeting of either the mutated PACS1 protein for proteasomal degradation by recruiting an E3 ubiquitin ligase or the mutated protein more directly, need the elucidation of the 3D structure of the PACS1 protein and the differences between the wild-type and the mutated forms. 

The review by Yang et al. [31] focuses on Ectodysplasin A (EDA) signaling, which regulates ectodermal development in all vertebrates. This core pathway comprising the three main proteins EDA, a member of the tumor necrosis factor (TNF-α) family of ligands, EDAR, the EDA receptor, and the recruited intracellular adaptor EDARADD (EDAR-associated death domain), relies on downstream NF-κB pathway activation to regulate target genes. Derangement of the EDA signaling by inactivating mutations affecting any of its players, leads to hypohidrosis ectodermal dysplasia (HED), a disorder characterized by impaired development of teeth (oligodontia), hair follicles (oligotrichosis) and exocrine sweat glands (oligohidrosis/anhidrosis,) with several subtypes showing autosomal dominant, autosomal recessive and X-linked inheritance patterns. The authors review studies on mouse and dog models, demonstrating that the postpartum and prenatal administration of the recombinant protein, consisting of the receptor-binding portion of EDA-A1 and the Fc domain of the immunoglobulin G1, ameliorates the disease phenotype. Results obtained using a fully humanized EDA-A1 protein injected via ultrasound-guided amniocentesis into an X-L HED fetus at 26 and 31 weeks of gestation determined a full recovery of sweat gland development and sweating ability [32], featuring recombinant EDA as the most promising therapeutics for HED. Alteration of the EDA pathway is also implicated in common diseases such as non-alcoholic fatty liver disease (NAFLD), type II diabetes and many cancer types. 
